# Efficacy and Safety of *Lactobacillus reuteri* CCFM1040 in Allergic Rhinitis and Asthma: A Randomized, Placebo-Controlled Trial

**DOI:** 10.3389/fnut.2022.862934

**Published:** 2022-04-07

**Authors:** Lingzhi Li, Zhifeng Fang, Yuan-kun Lee, Jianxin Zhao, Hao Zhang, Huaiming Peng, Yulong Zhang, Wenwei Lu, Wei Chen

**Affiliations:** ^1^State Key Laboratory of Food Science and Technology, Jiangnan University, Wuxi, China; ^2^School of Food Science and Technology, Jiangnan University, Wuxi, China; ^3^Department of Microbiology and Immunology, Yong Loo Lin School of Medicine, National University of Singapore, Queenstown, Singapore; ^4^National Engineering Research Center for Functional Food, Jiangnan University, Wuxi, China; ^5^Yancheng Sixth People's Hospital, Yancheng, China

**Keywords:** *Lactobacillus reuteri* CCFM1040, allergic rhinitis, asthma, clinical symptoms, safety, gut microbiota

## Abstract

The coexistence of allergic rhinitis (AR) and asthma reinforces the concept of “one airway, one disease,” which has prompted the exploration for a single intervention to treat both diseases. *Lactobacillus reuteri* CCFM1040 (CCFM1040) was found to be an inhibitor of the common pathogenesis of AR and asthma in our previous studies. This study presented a randomized, placebo-controlled trial to investigate the clinical effects of CCFM1040 on both diseases. The total symptom score (TSS), the quality of life (QoL), and the modulation in the gut microbiota of patients with AR, the Asthma Control and Test (ACT) of patients with asthma, and the safety of both AR and asthma were measured. In patients with AR, CCFM1040 numerically decreased TSS, Rhinoconjunctivitis Quality of Life Questionnaire (RQLQ), 3 nasal scores in TSS (nasal congestion, watery eyes, and rhinorrhea), and sleep and significantly improved (*P* = 0.014) non-nose/eye symptoms. The ACT score was numerically increased in patients with asthma (from partially controlled to well-controlled). Significant microbial (from class level to genus level) and metabolic differences (*P* < 0.05) were found in patients with AR. No adverse reactions were observed. No effect on the blood and urine routine indexes. CCFM1040 has a potential benefit on both diseases. Further studies based on these findings will help to optimize the management of AR and asthma.

## Introduction

Allergic rhinitis (AR) and asthma are the commonest chronic inflammatory disorders of the airways worldwide and can affect people of any age, impairing quality of life (QoL) (1, 2). Currently, AR and asthma are diagnosed and treated separately and have been treated only with symptomatic medications that have side effects (2). In 2002, Linneberg et al. reported that AR coexists in up to 80% of all patients with asthma (3). Although the underlying causes of AR and asthma are not fully understood, they share many pathophysiological links; in particular, both are characterized by type 2 immune inflammation (4, 5). Thereby, the concept of “one airway, one disease” has been emphasized in many pathophysiological, epidemiological, and clinical studies (2). Based on this concept, there might be a single intervention to treat both diseases, which is an exciting but unknown exploration.

Recently, the gut microbiota has been attracting attention. It is involved in homeostatic functions such as development, immune system activation, and nutrient absorption which are the basis for good health. The perturbation of gut microbiota could impair homeostatic functions, which are assumed to be responsible for the onset of inflammation (6, 7). This repercussion affected not only the health of the intestines but also the health of other organs in the human body, such as the respiratory tract. Moreover, the respiratory and intestinal tracts have structural similarities, and the microbiota in the respiratory and intestinal tract can communicate *via* aspiration (8). Hence, the gut microbiota plays a pivotal role in the etiology of AR and asthma; nevertheless, the exact mode of action of the gut microbiota is poorly understood. Notably, the gut microbiota is highly dynamic, forming microbial communities that are mainly influenced by gene and microbial exposure (9, 10). In recent decades, industrialization and urbanization have significantly reduced the agricultural microbial exposure, resulting in these microbes being missing in the intestine, which in turn greatly alter the gut microbiota structure and functional patterns of the human body, which ultimately lead to the development of AR and asthma (11, 12). It is suggested that supplementation with the “missing” agricultural microbes may represent a promising therapeutic strategy for covering AR and asthma.

Given the limited sequencing technology currently available, the intestinal microbes are only identified at the genus level. *Lactobacillus* (*L*.) was found to be significantly reduced in the intestines of individuals with both AR and asthma (13). Thus, supplementation with *Lactobacillus* would be a potential therapeutic modality for covering AR and asthma. Recently, their clinical benefits have emerged in AR or asthma populations, such as *Lactobacillus rhamnosus* GG inhibited the development in high risk of asthma in children and *Lactobacillus gasseri* A5 improved the symptoms in asthmatic children with AR (14, 15). However, the role of *Lactobacillus* in the treatment of AR or asthma has not been established, much less which covers both diseases. *Lactobacillus reuteri* is supposed to be a well-colonized bacteria species in the human intestine and can reduce multi-organ inflammation (16, 17). Moreover, the advantage of *L. reuteri* in reducing airway inflammation is more obvious than those of other *Lactobacillus* species in mice with asthma in our large-scale study (18). Mechanically, *L. reuteri* could modulate gut microbiota and metabolize endogenous tryptophan to balance systemic and mucosal immune reactivity, which is a key to benefit the homeostatic functions, thereby inhibiting airway inflammation (17, 19, 20). In our previous study, *L. reuteri* CCFM1040 (CCFM1040) was isolated from agricultural unpasteurized milk. The effect of CCFM1040 on targets with type 2 immune inflammation and gut microbiota was identified in mice (18, 21). Thus, supplementation with CCFM1040 would allow for effective intervention that potentially covers AR and asthma.

In this randomized, placebo-controlled trial, we aimed to investigate the effects of CCFM1040 on the clinical symptoms, QoL, modulation of gut microbiota and metabolism, and safety in adult patients with AR, AR with asthma, and asthma.

## Materials and Methods

### Study Design

This is a randomized, placebo-controlled study that evaluated the efficacy, safety, and gut microbiota modulation ability of CCFM1040 in patients with AR and/or asthma. The study was conducted according to the principles of the Declaration of Helsinki and all relevant local regulatory requirements. Yancheng Sixth People's Hospital Research Ethics Board granted written approval for the study. The ClinicalTrials.gov identifier of this study is ChiCTR1900024200. The number of the ethics committee approval is ET2019026. This study was conducted at the Yancheng Sixth People's Hospital, China.

### Study Sample

We recruited patients aged 18–60 years with at least a 1-year-long documented history of AR and/or asthma from August to October 2019 (a total of 12 weeks, including 4 weeks of recruitment and 8 weeks of intervention). Patients with a 1-month history of antibiotics use, pregnancy, and breastfeeding were excluded. The informed consent form approved by the Research Ethics Committee was signed by all the participants before starting any study procedure.

### Intervention

**Figure 1:**
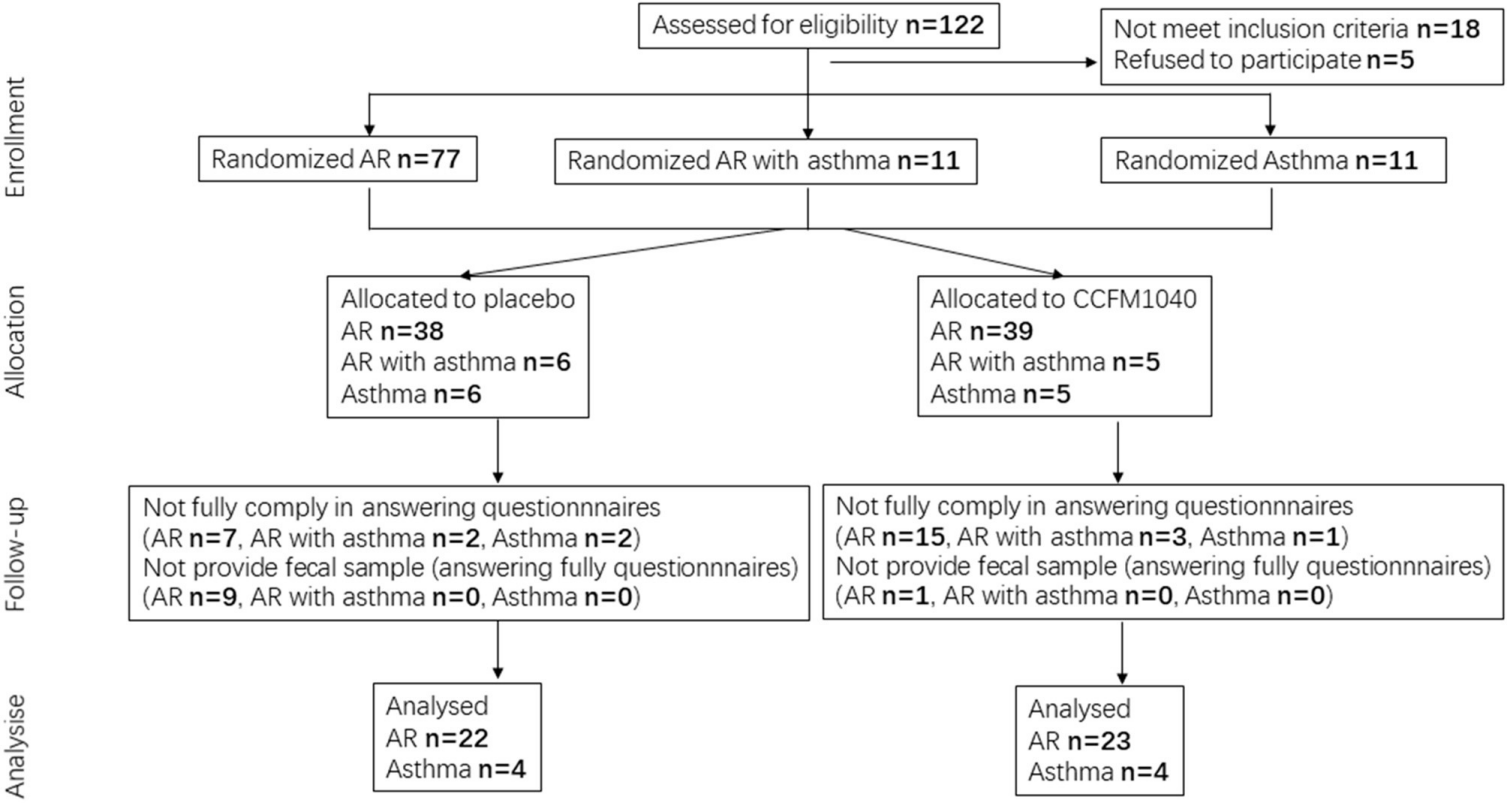
Flowchart of the study.

The patients were randomized at a ratio of 1:1 to receive CCFM1040 or placebo, using computer-generated block randomization. The study flowchart is shown in Figure 1. Blood, urine, and fecal samples were collected from the patients in the morning at baseline and at the end of the study. Before treatment, the patient's liver and kidney function were tested. All interventions were administered over an 8-week period. In the CCFM1040 group, the patients consumed a freeze-dried bacteria product containing 10 (9) colony-forming units (CFU) and maltodextrin (excipients) per 2 g sachet once daily. In the placebo group, the patients received a product containing only maltodextrin once daily. CCFM1040 and placebo products had the same volume, size, taste, color, and shape and were dispensed by Jiangsu Weikang Biological Technology Limited Company (Suzhou, China). They were available in the form of an oral dispersible powder in sachets and were stable at room temperature for a month. Thus, the patients were given the required stock of CCFM1040 or placebo products two times (at baseline and week 4). The dispersible powder can be taken directly into the mouth or dissolved in warm water (<37°C) after a meal. During 8 weeks, the patients were requested not to take any other probiotic products.

### Outcome Assessment

The patients filled out self-report online questionnaires at baseline, the midpoint (week 4), and the end of the study. The total symptom score (TSS) form and the Rhinoconjunctivitis Quality of Life Questionnaire (RQLQ) were filled out by patients with AR and AR with asthma, and the Asthma Control and Test (ACT), by those with asthma and AR with asthma.

The TSS form measures both nasal symptoms (nasal congestion, itching, sneezing, and rhinorrhea) and eye symptoms (itchy/red eyes and watery eyes), which scored 0 (no symptoms), 1 (mild), 2 (moderate), or 3 (severe). These scores are summed to produce the TSS (range: 0–18).

The ACT measures the adequacy of asthma control and change in asthma control as reported by the patients: a score of 20–25 points denotes that asthma is well-controlled, 16–19 points, partially controlled asthma, and 5–15 points, uncontrolled asthma.

The RQLQ is a valid scale that assesses the QoL according to how the symptoms affect the patients with AR. It consists of 28 questions covering seven domains (activities, non-nose/eye symptoms, sleep, practical problems, nasal symptoms, eye symptoms, and emotions). The average score for all items ranges from 0 (not at all) to 6 (extremely).

### Sampling, DNA Extraction, and High-Flux Sequencing

The fecal samples were collected from individuals in a sterile tube, liquid nitrogen frozen, and stored at −80°C. DNA from the fecal sample was extracted using a Fast DNA SPIN Kit for Feces (MP Biomedicals; Carlsbad, CA, USA). The *16S* rRNA genes were amplified with specific primers (*341F* and *806R*) for the V3-V4 region. The amplicon libraries were pooled and subjected to sequencing on an Illumina MiSeq High-Flux Sequencing Platform (Illumina, San Diego, USA), which was described previously (22, 23).

### Bioinformatic Analyses

The analysis of the gut microbiota was performed using QIIME2 software. The dataset comprising 731,349 high-quality (8,126 counts per sample on average) classifiable read counts was generated after Illumina MiSeq sequencing of 90 samples. The number of counts in the samples ranged from 277 to 15,226. All data were subjected to total sum scaling normalization. The R phyloseq (24) and vegan packages (25) were used to analyze the community diversity profile. The data used for α-diversity analysis were not rarefied. The differences between the groups were analyzed by the Mann-Whitney *U*/Kruskal-Wallis (nonparametric) tests. The hierarchical structure of taxonomic classifications to the median abundance (quantitatively) and the nonparametric Wilcoxon Rank Sum test (statistically) was used to depict the taxonomic differences between microbial communities (26). PICRUSt was used to predict microbial functions (http://huttenhower.sph.harvard.edu/galaxy) (27).

### Safety

Blood routine and urine routine were detected before and after treatment. Adverse events were recorded at each visit.

### Statistical Methods

The data on rhinitis symptoms, asthma symptoms, and QoL were analyzed using GraphPad Prism version 8 (La Jolla, CA, USA) and SPSS version 22.0 (SPSS Inc., Chicago, IL, USA). The data are expressed as mean ± SEM. The differences between the groups were compared using the *t*-test and the analysis of covariance (ANCOVA). Both 95% CI and *P*-values were provided for differences between the CCFM1040 group and the placebo group.

## Results

### Baseline Demographic and Clinical Characteristics

A total of 99 patients with AR (77), AR with asthma (11), and asthma (11) were enrolled. All the patients were of Han nationality, had lived in their current residence for more than 1 year, and had a carbohydrate-based diet; 45 patients with AR completed the questionnaires and had their fecal samples analyzed, and eight patients with asthma completed the questionnaires (CONSORT flow diagram; Figure 1). For efficacy evaluation, we used the data of 45 patients with AR and eight patients with asthma. The characteristics of the placebo and CCFM1040 groups are summarized in Table 1. The baseline demographic and clinical characteristics of the patients with AR and asthma were similar between the groups.

**Table 1:** Demographic data.

**Characteristic**	**AR**		**Asthma**	
	**Placebo** **(*n =* 22)**	**CCFM1040** **(*n =* 23)**	**Placebo** **(*n =* 4)**	**CCFM1040** **(*n =* 4)**
Sex, no (%)				
Male	9 (40.9)	9 (39.1)	1 (25)	0 (0)
Age (y)				
Mean ± SEM	42.5 ± 10.7	44.8 ± 8.5	48.5 ± 6.8	41.0 ± 10.6
Range	18–58	31–60	42–58	30–58
Body mass index (kg/m^2^)				
Mean ± SEM	22.3 ± 3.6	23.4 ± 2.9	25.7 ± 3.2	22.6 ± 1.6
Range	17.4–30.9	17.9–27.6	21.8–30.4	20.8–25.1
Body mass index >30 kg/m^2^				
no (%)	1 (4.55)	0 (0)	1 (25)	0 (0)
Allergies run in the family				
no (%)	8 (36.4)	9 (39.1)	1 (25)	1 (25)
Smokers in household				
no (%)	4 (18.2)	4 (17.4)	1 (25)	0 (0)
Probiotics history, no (%)				
None	13 (59.1)	15 (65.2)	3 (75)	2 (50)
Past	7 (31.8)	7 (30.4)	1 (25)	1 (25)
Current	2 (9.1)	1 (4.3)	0 (0)	1 (25)
Childhood residence				
Rural	15 (68.2)	15 (65.2)	3 (75)	3 (75)
Urban	7 (31.8)	8 (34.8)	1 (25)	1 (25)
TSS				
Mean ± SEM	7.8 ± 1.0	8.0 ± 0.7	-	-
RQLQ				
Mean ± SEM	57.9 ± 8.3	56.0 ± 8.0	-	-
ATC				
Mean ± SEM	-	-	17 ± 1.7	19.5 ± 0.6

*AR, allergic rhinitis. TSS, total symptom score. ACT, asthma control test. RQLQ, Rhinoconjunctivitis Quality of Life Questionnaire*.

### Efficacy Outcome

**Figure 2:**
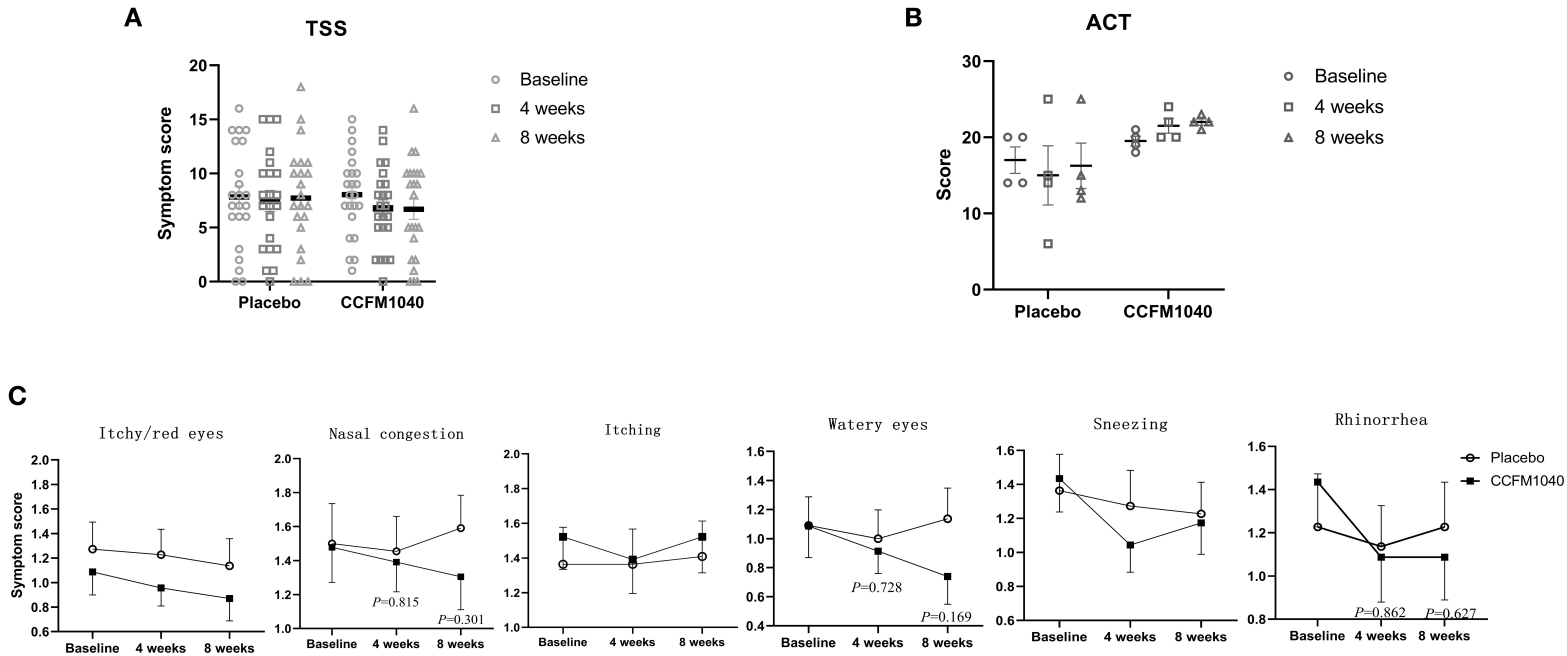
Clinical symptoms in CCFM1040 and placebo groups during the study period. Results in TSS of AR **(A)**, ACT of asthma **(B)**, and subgroups symptom scores of AR **(C)**. Data are expressed as mean (SEM). The differences between the groups were compared using the *t*-test, and the *P*-values are at a 5% level vs. placebo. AR, allergic rhinitis; TSS, total symptom score; ACT, asthma control test.

After 4 weeks of the intervention, the TSS of the patients with AR was lower in the CCFM1040 group than in the placebo group (mean difference, 0.672; 95%CI, −1.815 to 3.163), and this efficacy was magnified until the 8th week (1.032; 95%CI, −1.782 to 3.845) (Figure 2A). Furthermore, reductions in the subscales of the TSS form also occurred in the CCFM1040 group (Figure 2C). From week 4 to week 8, the improvement in score relative to placebo was (0.063; 95%CI,−0.479 to 0.605; *P* = 0.815 vs. placebo) to (0.287; 95%CI, −0.265 to 0.838; *P* = 0.301 vs. placebo) for nasal congestion, (0.087; 95%CI, −0.414 to 0.588; *P* = 0.728 vs. placebo) to (0.397; 95%CI, −1.176 to 0.970; *P* = 0.169 vs. placebo) for watery eyes, and (0.049; 95%CI, −0.519 to 0.618; *P* = 0.862 vs. placebo) to (0.140; 95%CI, −0.437 to 0.718; *P* = 0.627 vs. placebo) for rhinorrhea. A higher reduction in mean sneezing score was observed in the CCFM1040 group than in the placebo group after 4 weeks of intervention (0.229; 95%CI, −0.300 to 0.759; *P* = 0.387 vs. placebo), while a recovery was observed until 8 weeks (0.053; 95%CI, −0.475–0.512; *P* = 0.840 vs. placebo). For itchy/red eyes and itching, no differences relative to placebo were observed in the CCFM1040 group.

The ACT scores of asthmatic patients confirmed the findings of the primary efficacy analysis, showing an increase in the CCFM1040 group (from partially controlled to well-controlled) than a decrease in the placebo group during 8 weeks of the intervention (−5.75; 95%CI, −13.116 to 1.616; *P* = 0.105 vs. placebo) (Figure 2B).

**Figure 3:**
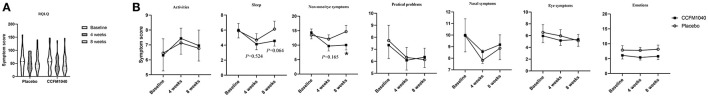
QoL of AR in CCFM1040 and placebo groups during the study period. Results in RQLQ **(A)** and subgroup QoL scores **(B)**. Data are expressed as mean (SEM). The differences between the groups were compared using the *t*-test. RQLQ, sleep, non-nose/eye symptom, and eye symptoms are based on the analysis of covariance (ANCOVA), with the midpoint and the end results as the dependent variables, group as the fixed factors, and baseline as the covariates; *P*-values are from the two-sided test at a 5% level vs. placebo. AR, allergic rhinitis; QoL, quality of life; RQLQ, Rhinoconjunctivitis Quality of Life Questionnaire.

The improvements in RQLQ during the 8-week intervention kept dropping in the CCFM1040 group than in the placebo group from (4.416; 95%CI, −8.875 to 17.207; *P* = 0.506 vs. placebo) to (6.399; 95%CI, −7.741 to 20.593; *P* = 0.366 vs. placebo) (Figure 3A). Regarding the RQLQ subscales, no consistent improvement in activities, practical problems, nasal symptoms and eye symptoms, and emotions occurred at any study point. Notably, reductions in the CCFM1040 group were significantly greater than those in the placebo group for non-nose/eye symptoms (2.750; 95%CI, −1.181 to 6.682; *P* = 0.165 vs. placebo; week 4) and (5.180; 95%CI, −1.120 to 9.329; *P* = 0.014 vs. placebo; week 8) and were greater for sleep (0.592; 95%CI, −1.266 to 2.451; *P* = 0.524 vs. placebo; week 4) and (1.629; 95%CI, −0.010 to 3.358; *P* = 0.064 vs. placebo; week 8) across the 8-week intervention period (Figure 3B).

### Gut Microbiota Profiles in Patients With AR Were Different After CCFM1040 Intervention

**Figure 4:**
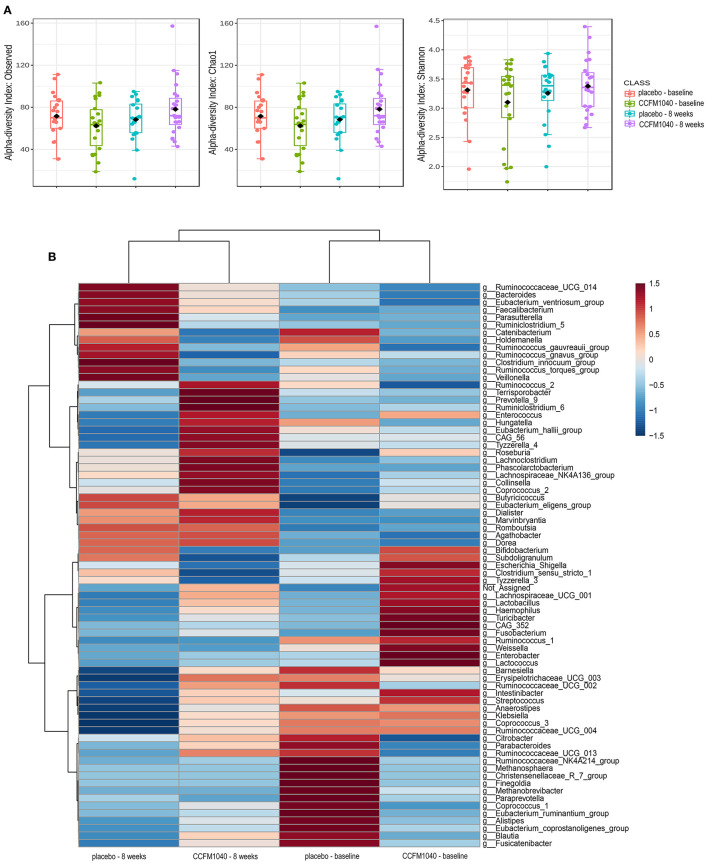
Effect of CCFM1040 and placebo on the composition of the gut microbiota in patients with AR at the genus level. *P* < 0.05. **(A)** Boxplot for α-diversity measured based on observed, Chao1, and Shannon indices. **(B)** Heatmap analysis of species abundance clustering. The heatmap shows the rank based on abundance. Each column represents one group, while each row in the heatmap represents one genus. The red to blue color bar indicates the relative abundance. AR, allergic rhinitis.

**Figure 5:**
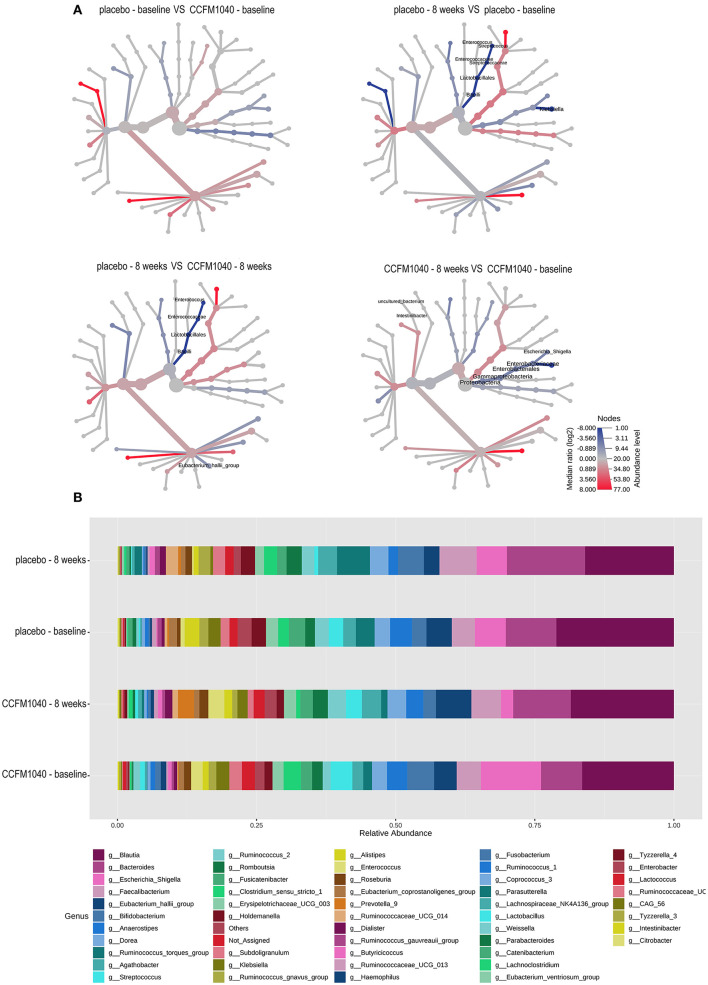
Effect of CCFM1040 and placebo on the composition of gut microbiota in patients with AR. **(A)** The different abundance of microbe between the two groups was shown in the heat tree. Nodes marked with text represent significance (*P* < 0.05) between the two groups. **(B)** Effect of CCFM1040 and placebo on the abundance of the bacterial genus. AR, allergic rhinitis.

The baseline gut microbiota profile as per *16S* rRNA gene sequencing was not significantly (*P* > 0.05) different between the groups (**Figure 5**). After an 8-week intervention, the influence of CCFM1040 in the gut microbiota was analyzed. The α-diversity indices were examined to analyze the microbial diversity (Figure 4A). The Observed, Chao1, and Shannon Index metrics were numerically increased but not significantly (*P* > 0.05) in the CCFM1040 group compared with the placebo group. The hierarchical clustering of the heatmap revealed significant alterations (*P* < 0.05) from the class level to the feature level, which resulted in distinct clustering of microbes in the CCFM1040 group and the placebo group (Figure 4B and Supplementary Figures 1–4). It is suggested that the intervention of both CCFM1040 and placebo formatted a new gut microbial structure. Then, heat tree analysis identified the altered microbes in different groups (Figure 5). No significant difference (*P* > 0.05) was found between the two groups before the intervention. After the intervention, CCFM1040 and placebo groups both altered some of the abundances of the microbes, but they were completely different. The gut bacteria from the phylum Proteobacteria to the genus *Escherichia_Shigella* and the order Intestinibacter were significantly decreased in the CCFM1040 group compared with the baseline, whereas the placebo markedly decreased the abundance of the gut microbes under the class Bacilli compared with both the baseline and the CCFM1040 group. These results indicated that CCFM1040 modulated the gut microbiota structure in a fundamentally different way compared with the placebo in patients with AR.

### Patients With AR in the CCFM1040 Group Improved the Function of Gut Microbiota

**Figure 6:**
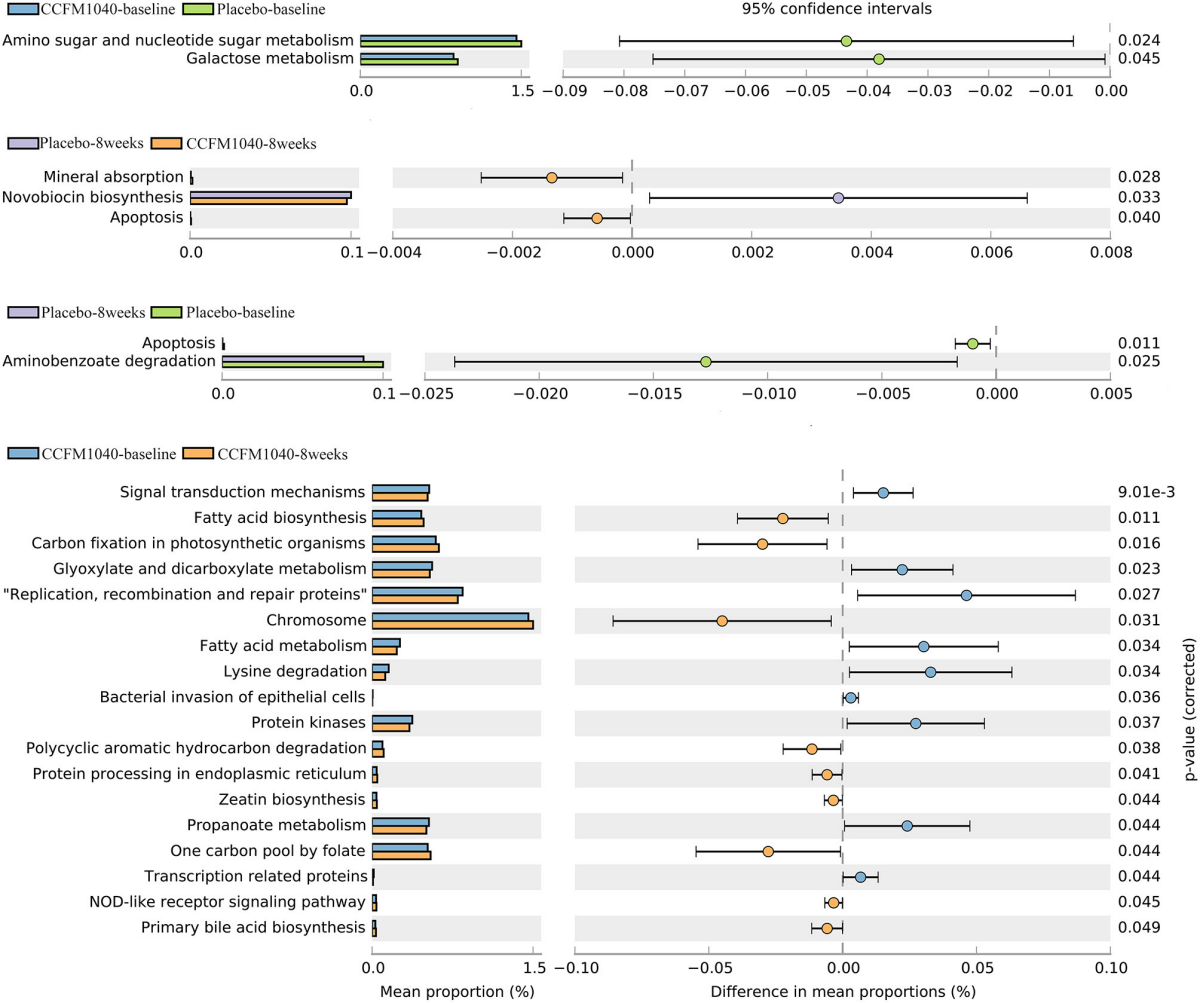
Effect of CCFM1040 on the metabolism of the gut microbiota in patients with AR. *P* < 0.05. AR, allergic rhinitis.

To characterize the functional composition profiles after CCFM1040 intervention, we predicted the gut microbial functions. A total of 328 biological pathways were identified. As shown in Figure 6, after filtering out the pathways associated with human diseases, plant functions, drug development, and organismal systems, (28) the distinct patterns of metabolism were revealed in the CCFM1040 and placebo-supplemented groups. Mineral absorption and apoptosis were significantly promoted (*P* < 0.05), and the novobiocin biosynthesis was dramatically inhibited (*P* < 0.05) after the CCFM1040 intervention compared with the placebo. Meanwhile, compared with the baseline, CCFM1040 significantly altered (*P* < 0.05) 18 biological pathways, which were mainly associated with carbohydrate, energy and lipid metabolisms, xenobiotic biodegradation and metabolism, metabolism of cofactors and vitamins, and immune system. Placebo, however, altered only two metabolic pathways: apoptosis and aminobenzoate degradation as compared with before the intervention.

### Safety/Adverse Events

CCFM1040 treatment was well-tolerated, and no side effects or adverse events were recorded. After the 8-week intervention, blood and urine specimens were provided by more than 80% of the patients. There was no significant impact of the intervention on the patients' blood and urine routine (Table 2).

**Table 2:** Blood and urine routine.

**Index**	**AR**				**Asthma**			
	**Placebo**		**CCFM1040**		**Placebo**		**CCFM1040**	
	**Before**	**After**	**Before**	**After**	**Before**	**After**	**Before**	**After**
**Blood mean (range)**	***n =*** **20**		***n =*** **20**		***n =*** **4**		***n =*** **3**	
Red blood cell	4.60 (3.52–5.57)	4.62 (3.76–5.33)	4.62 (3.9–5.67)	4.65 (3.94–5.34)	4.38 (4.04–4.82)	4.32 (4.11–4.61)	4.62 (4.09–4.98)	4.46 (3.96–5.04)
Hemoglobin concentration	134 (92–173)	139 (110–163)	136 (112–166)	138 (113–164)	124 (101–137)	127(103–145)	142 (123–165)	130 (145–119)
Platelets	229 (102–361)	240 (149–389)	223 (131–338)	239 (157–357)	237 (179–322)	237 (195–297)	207 (164–278)	172 (146–186)
Leukocyte	5.07 (2.88–6.63)	5.80 (4.09–8.01)	5.63 (4.39–7.22)	5.78 (3.63–8.33)	6.30 (4.48–8.74)	6.85 (4.92–8.59)	5.67 (3.67–8.93)	4.86 (4.23–6.07)
Glucose	5.01 (4.18–5.89)	4.95 (3.19–5.83)	5.18 (4.03–7.65)	5.09 (4.27–6.00)	4.94 (4.66–5.26)	5.32 (4.93–6.31)	5.56 (4.83–6.52)	5.94 (4.92–7.73)
Total cholesterol	4.49 (3.56–6.08)	4.46 (3.25–5.95)	4.74 (3.39–6.74)	5.13 (3.71–7.08)	4.71 (3.95–5.74)	5.30 (4.23–5.69)	6.13 (5.66–6.95)	6.22 (5.3–6.69)
High density lipoprotein cholesterol	1.53 (1.10–1.93)	1.42 (0.86–2.14)	1.51 (1.08–2.12)	1.45(0.95–1.97)	1.60 (1.17–2.02)	1.56 (1.06–2.02)	1.66 (1.63–1.69)	1.74 (1.64–1.88)
Low density lipoprotein cholesterol	2.63 (1.68–4.26)	2.72 (1.72–4.36)	2.90 (1.74–4.84)	3.34 (2.12–5.29)	2.82 (2.26–3.29)	3.13 (2.40–3.62)	3.19 (1.96–4.01)	4.01(3–4.86)
**Urine**	***n =*** **17**		***n =*** **14**		***n =*** **4**		***n =*** **3**	
Positive of urine sugar no (%)	0 (0)	0 (0)	1 (7.14)	0 (0)	0 (0)	0 (0)	0 (0)	0 (0)
Specific gravity Mean (range)	1.02 (1.01–1.02)	1.02 (1.02–1.02)	1.02 (1.01–1.02)	1.02 (1.01–1.02)	1.02 (1.02–1.02)	1.02 (1.02–1.02)	1.02 (1.02–1.02)	1.02 (1.02–1.02)
pH mean (range)	5.91 (5.00–7.00)	6.41 (6.00–8.50)	6.11 (5.50–7.00)	6.25 (5.50–7.00)	5.75 (5.50–6.00)	6.38 (6.00–7.00)	6.00 (6.00–6.00)	6.00 (6.00–6.00)
Positive of urine protein no (%)	1 (5.88)	2 (11.76)	0 (0)	1 (7.14)	1 (25)	0 (0)	0 (0)	0 (0)
Positive of urobilinogen no (%)	0 (0)	0 (0)	0 (0)	0 (0)	0 (0)	0 (0)	0 (0)	0 (0)
Positive of nitrite no (%)	0 (0)	0 (0)	0 (0)	0 (0)	0 (0)	0 (0)	0 (0)	0 (0)
Positive of leucocyte esterase no (%)	3 (17.65)	3 (17.65)	1 (7.14)	1 (7.14)	2 (50)	2 (50)	2 (66.67)	1 (33.33)

*AR, allergic rhinitis*.

## Discussion

Both AR and asthma have been emphasized the “one airway, one disease,” because they share similar type 2 inflammation. It is exciting but unknown to explore a single intervention to treat both diseases. Previously, we had conducted animal experiments on CCFM1040 and demonstrated its favorable effect on targets with type 2 inflammation by modulating gut microbiota and metabolism. In this study, we investigated the beneficial effect of CCFM1040 on patients with both AR and asthma. To the best of our knowledge, this is the first study that reported the simultaneous effect of *Lactobacillus* on both diseases in adult patients.

Our data showed that CCFM1040 not only improves symptoms and the QoL in patients with AR but also strengthens the control in patients with asthma. This is an important finding because it supports the hypothesis that supplementation with one single intervention, such as the “missing” agricultural microbes, might be used to treat AR and asthma simultaneously.

Currently, there are no accepted criteria for determining the optimal parameters from regulatory authorities or academia for clinical microbiological intervention trials. In general, patient-rated TSS and RQLQ are preferred as a primary measure of efficacy in AR trials for drug development, but some researchers reported that RQLQ may insufficiently or inaccurately capture the efficacy of anti-AR drugs (29, 30). For evaluating asthma improvement, the ACT is generally used. Previously, some clinical trials have evaluated the efficacy of microbial strains in AR by using the TSS, and some studies by using the RQLQ. This makes it difficult to analyze the outcomes of different clinical trials. Therefore, in this study, simultaneous observation of TSS, RQLQ, and ACT is advantageous. Moreover, the results showed that TSS, watery eyes (TSS), nasal congestion (TSS), rhinorrhea (TSS), RQLQ, sleep (RQLQ), and ACT showed a descending tendency, and non-nose/eye symptoms (RQLQ) were significantly improved (*P* < 0.05), which suggest that these indicators have adequate sensitivity in the microbiological intervention trial.

The multiple causes of AR and asthma in patients complicate the management of these conditions (2). Based on a similar type 2 inflammation that occurs in AR and asthma, the possibility of using one single intervention to treat both diseases was proposed in this study. In mice with allergic asthma, we found that CCFM1040 clearly reduced the widespread type 2 inflammation by upregulating the population of regulatory T cells (Treg cells) (21). Treg cells are involved in systemic immunity that simultaneously regulates multiple immunological processes. Due to CCFM1040 affecting systemic immunity, we speculated that it can simultaneously benefit AR and asthma in humans. The results support our speculation, and the effects of CCFM1040 on clinical symptoms might be related to its immunoregulation. It is an indication that further study requires more attention to the mechanism by which microbes treat both diseases through modulating systemic immunity.

The AR is a highly prevalent but often an underdiagnosed, untreated, or undertreated chronic disease (31, 32). If left untreated or ineffectively treated, AR symptoms can have a negative influence on sleep, which can negatively impact productivity, sport and leisure activities, academics, and health-related QoL (33, 34). Generally, AR symptoms precede the onset of asthma, and the successful treatment of AR controls the attacks and exacerbations of asthma. In our study, CCFM1040 improved sleep and non-nose/eye symptoms (*P* < 0.05) in patients with AR. This is consistent with the previous results: microorganisms can improve certain indicators of AR (35, 36). Therefore, the effectiveness of regular ingestion of CCFM1040 on these indicators of AR and the mechanism involved is worthy of further research, which may facilitate the founding of targeted therapies.

In general, the beneficial effects of the microbes and drugs on AR or asthma have been observed 4 weeks after continuous administration (35). In this study, the CCFM1040 intervention was designed to last for 8 weeks. The results showed that some scores of rhinitis, asthma symptoms, and QoL of the patients tended to decrease at week 4 and continued to decrease at week 8. It is suggested that further study should take longer to analyze the beneficial effects of CCFM1040 on both diseases. Nevertheless, similar to other studies, (36, 37) our study showed a clear improving trend in the clinical symptoms over a short period.

The important role of supplementation with the “missing” agricultural microbes in the treatment of AR and asthma was emphasized in this study, but their action on gut microbiota is still not clear. The patients with AR and asthma have low total diversity (including the decrease of *Lactobacillus*) and altered composition of the gut microbiota (13). Some studies have demonstrated that supplementation with *Lactobacillus* could rescue abnormal gut microbiota. In this study, we evaluated the effect of the CCFM1040 supplement on the gut microbiota of patients with AR. The results showed that the diversity of the gut microbiota in the CCFM1040-intervention group was higher after the intervention than the placebo. Unsurprisingly, the composition of the gut microbiota in the CCFM1040 group was different from the placebo intervention. Of note, *Enterococcus* and *Streptococcus*, which diminished in the placebo group, are widely recognized as beneficial bacteria (38) that produce butyrate and have shown anti-inflammatory effects in an animal model with airway inflammatory (39), indicating their positive association with the reduced risk of AR and asthma. Considering this point, the modulation in the placebo group might have had a negative impact. In contrast, microorganisms from the phylum Proteobacteria diminished in the CCFM1040 group, which are considered common factors in human diseases and show enrichment in the gut of children with rhinitis (40). Hence, the higher diversity of gut microbes, the enrichment of beneficial bacteria, and the weakening of harmful gut microbes in the CCFM1040 group might be a key to the beneficial effects of its intervention. However, it cannot be ignored that supplementation with intestinal microbes can alter the activity of resident bacteria and their interaction with the host (41), and certain bacteria are inherently the immune modulators (42), which are also related to the improvement of symptoms. As such, the modulation of the gut microbiome is a complex process. Therefore, further studies are needed to address these complex relationships.

The gut microbiota also produces small-molecule metabolites that enter the circulation to interact with the host. The metabolites produced in individuals with AR and asthma are quite different from those produced in healthy individuals (43). CCFM1040 directly modulates the gut microbiota in patients with AR, which is bound to affect the metabolic pathway and metabolites. In mice, the mechanism of action of this strain was explained as a role in modulating tryptophan metabolism in the intestine. However, the significant effect on modulating tryptophan metabolism has not appeared in humans as it does in mice. A major reason should be that the data in the previous animal study are based on the metabolomics methodology. Surprisingly, compared to the data in the previous animal study that are based on the predictive analysis of *16S* rRNA sequencing data, CCFM1040 caused alterations in more metabolic pathways of the gut microbiota, suggesting that the impact of CCFM1040 on the metabolic patterns of the gut microbiota in humans is greater than those that were observed in mice. Previously, children with asthma experienced alter in carbohydrate and lipid metabolism when treated with budesonide (44). Therefore, the alteration in carbohydrate and lipid metabolic functions may be a positive factor that CCFM1040 could help alleviate AR symptoms. Additionally, energy metabolism, (45) biodegradation and metabolism of xenobiotics, (46) metabolism of cofactors and vitamins (47), and the immune system are reported to protect against AR and asthma. Based on the abovementioned factors, we strongly believe that the CCFM1040-based modulation of gut metabolic patterns plays a crucial role in airway inflammation disease. However, the direct role of these metabolic patterns of the gut microbiota after CCFM1040 intervention remains unclear, and further functional analyses are needed.

*Lactobacillus* is generally beneficial and safe for healthy individuals but may cause adverse effects on some patients (48). For example, a significant increase in the incidence of bacteremia has been reported in patients in the intensive care unit when they are treated with *Lactobacillus*. Considering this point, we monitored the adverse effects and analyzed the health indicator. The data indicate that CCFM1040 has an acceptable safety profile and is well-tolerated in patients with AR and asthma.

There are also some limitations to this trial. The sample size is not large enough, even if larger than several previous trials with *Lactobacillus* intervention in AR or asthma. In a short period (4 weeks), sufficient patients with AR were recruited, while few with asthma and AR with asthma only were recruited. During the study, our patients were not comprehensive enough when filling out the online questionnaires and were reluctant to provide stool samples. This had a major impact on the comparison between CCFM1040 and placebo due to a drastic decrease (42%) in the number of samples we could analyze (Figure 1). Further research should be systematic in terms of online questionnaire completion and sufficient encouragement in terms of stool sample collection. Another limitation is that the duration of improvement in the CCFM1040 group was not observed further after the intervention. As such, these results could serve as the basis for prospective clinical trials involving larger numbers of patients and longer time.

## Conclusion

The randomized, placebo-controlled study pioneered the simultaneous effect of *Lactobacillus* on both AR and asthma in adult patients. In this trial, CCFM1040 showed potential benefits for patients with both AR and asthma. These benefits may be associated with its ability to modulate gut microbiota. CCFM1040 was also generally well-tolerated and safe in the overall study population. These data indicated that CCFM1040 has a potential role in simultaneously treating AR and asthma and warrants further large-scale clinical trials to validate the results.

## Data Availability Statement

The data presented in the study are deposited in the NCBI repository, accession number PRJNA807120. http://www.ncbi.nlm.nih.gov/bioproject/807120.

## Ethics Statement

The studies involving human participants were reviewed and approved by Yancheng Sixth People's Hospital Research Ethics Board. The patients/participants provided their written informed consent to participate in this study.

## Author Contributions

LL: investigation, methodology, data curation, visualization, writing—original draft, and writing—review and editing. ZF: data curation and visualization. Y-kL: supervision and resources. JZ: funding acquisition, project administration, and supervision. HZ: conceptualization and resources. HP and YZ: supervision and validation. WL: funding acquisition, writing—original draft, and writing—review and editing. WC: supervision, funding acquisition, project administration, and resources. All authors contributed to the article and approved the submitted version.

## Funding

This study was supported by the National Natural Science Foundation of China (Grant No. 31820103010) and the Collaborative Innovation Center of Food Safety and Quality Control in Jiangsu Province, and the 111 Project (Grant No. BP0719028).

## Conflict of Interest

The authors declare that the research was conducted in the absence of any commercial or financial relationships that could be construed as a potential conflict of interest.

## Publisher's Note

All claims expressed in this article are solely those of the authors and do not necessarily represent those of their affiliated organizations, or those of the publisher, the editors and the reviewers. Any product that may be evaluated in this article, or claim that may be made by its manufacturer, is not guaranteed or endorsed by the publisher.

## Supplementary Material

Supplementary Figure 1 Heatmap analysis of class abundance clustering in patients with AR. *P* < 0.05. The heatmap shows the rank based on abundance. Each column represents one group, while each row in the heatmap represents one class. The red to blue color bar indicates the relative abundance. AR, allergic rhinitis.

Supplementary Figure 2 Heatmap analysis of order abundance clustering in patients with AR. *P* < 0.05. The heatmap shows the rank based on abundance. Each column represents one group, while each row in the heatmap represents one order. The red to blue color bar indicates the relative abundance. AR, allergic rhinitis.

Supplementary Figure 3 Heatmap analysis of family abundance clustering in patients with AR. *P* < 0.05. The heatmap shows the rank based on abundance. Each column represents one group, while each row in the heatmap represents one family. The red to blue color bar indicates the relative abundance. AR, allergic rhinitis.

Supplementary Figure 4 Heatmap analysis of feature abundance clustering in patients with AR. *P* < 0.05. The heatmap shows the rank based on abundance. Each column represents one group, while each row in the heatmap represents one feature. The red to blue color bar indicates the relative abundance. AR, allergic rhinitis.
