# Supplementary figures and images for: Efficacy and Safety of Lactobacillus reuteri CCFM1040 in Allergic Rhinitis and Asthma: A Randomized, Placebo-Controlled Trial
Source: Front Nutr. 2022 Apr 7;9:862934. doi: 10.3389/fnut.2022.862934 (PMC9022948; doi:10.3389/fnut.2022.862934)

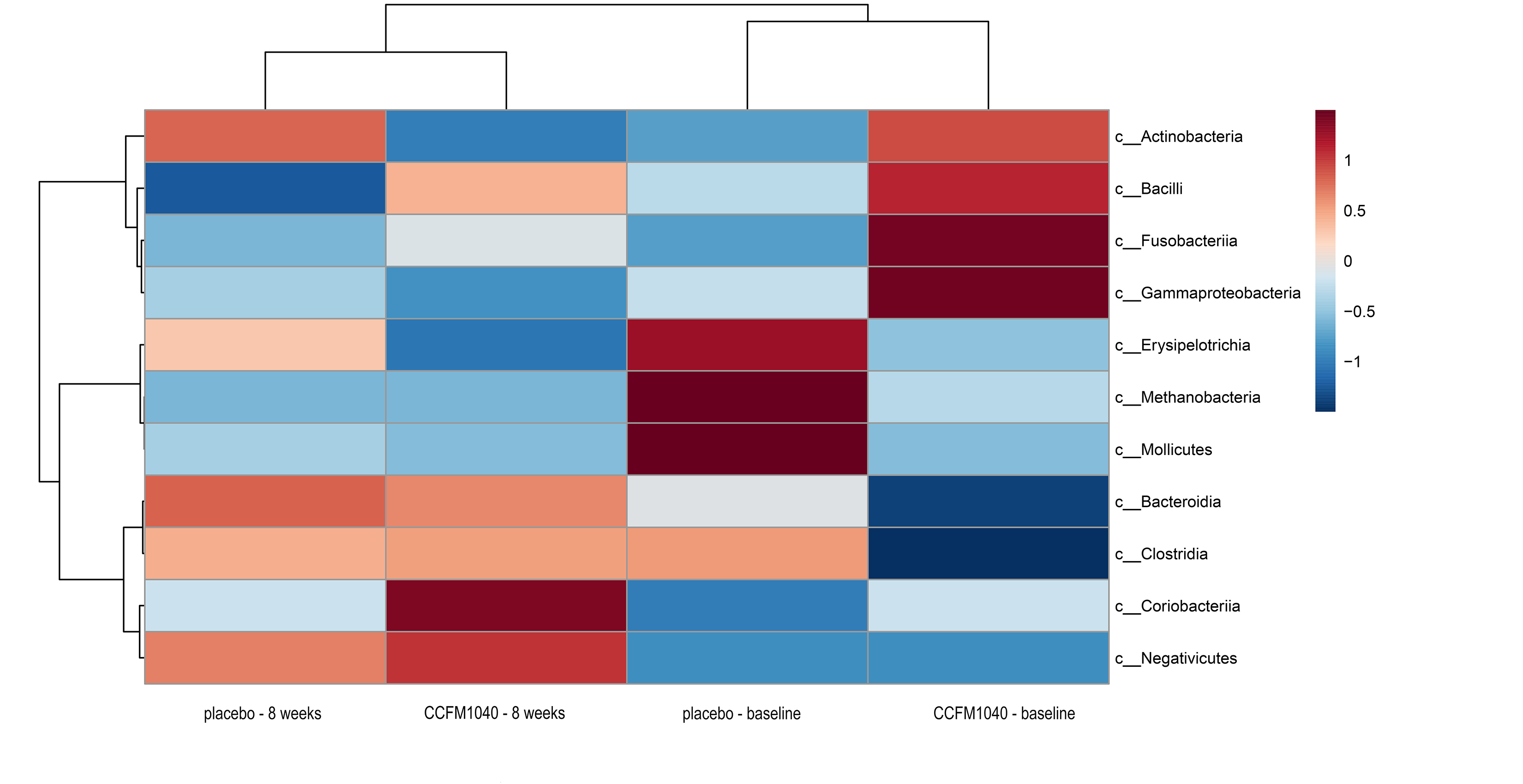

Supplement: Supplementary Figure 1 — Heatmap analysis of class abundance clustering in patients with AR. P < 0.05. The heatmap shows the rank based on abundance. Each column represents one group, while each row in the heatmap represents one class. The red to blue color bar indicates the relative abundance. AR, allergic rhinitis. [file Image_1.TIF]

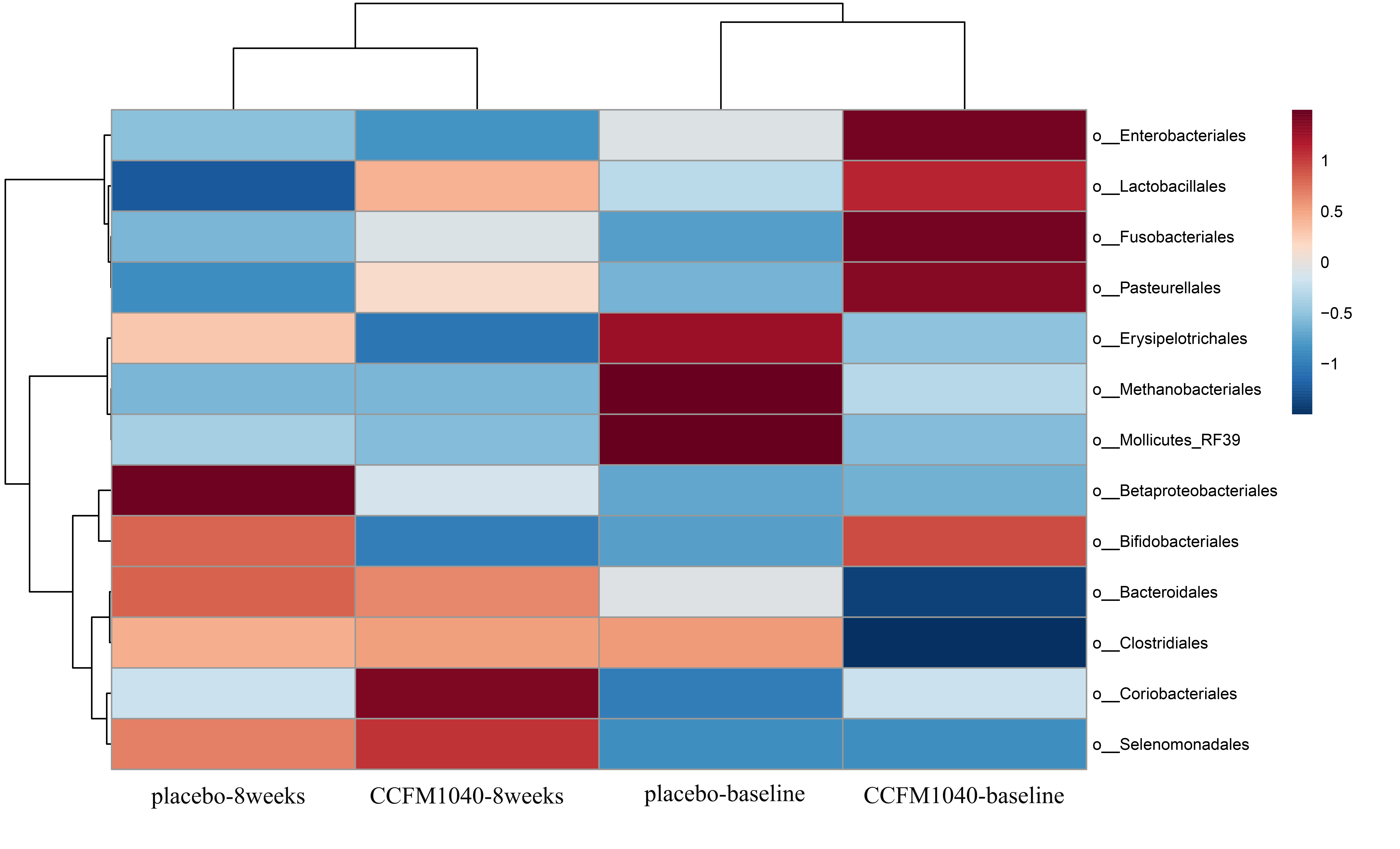

Supplement: Supplementary Figure 2 — Heatmap analysis of order abundance clustering in patients with AR. P < 0.05. The heatmap shows the rank based on abundance. Each column represents one group, while each row in the heatmap represents one order. The red to blue color bar indicates the relative abundance. AR, allergic rhinitis. [file Image_2.TIF]

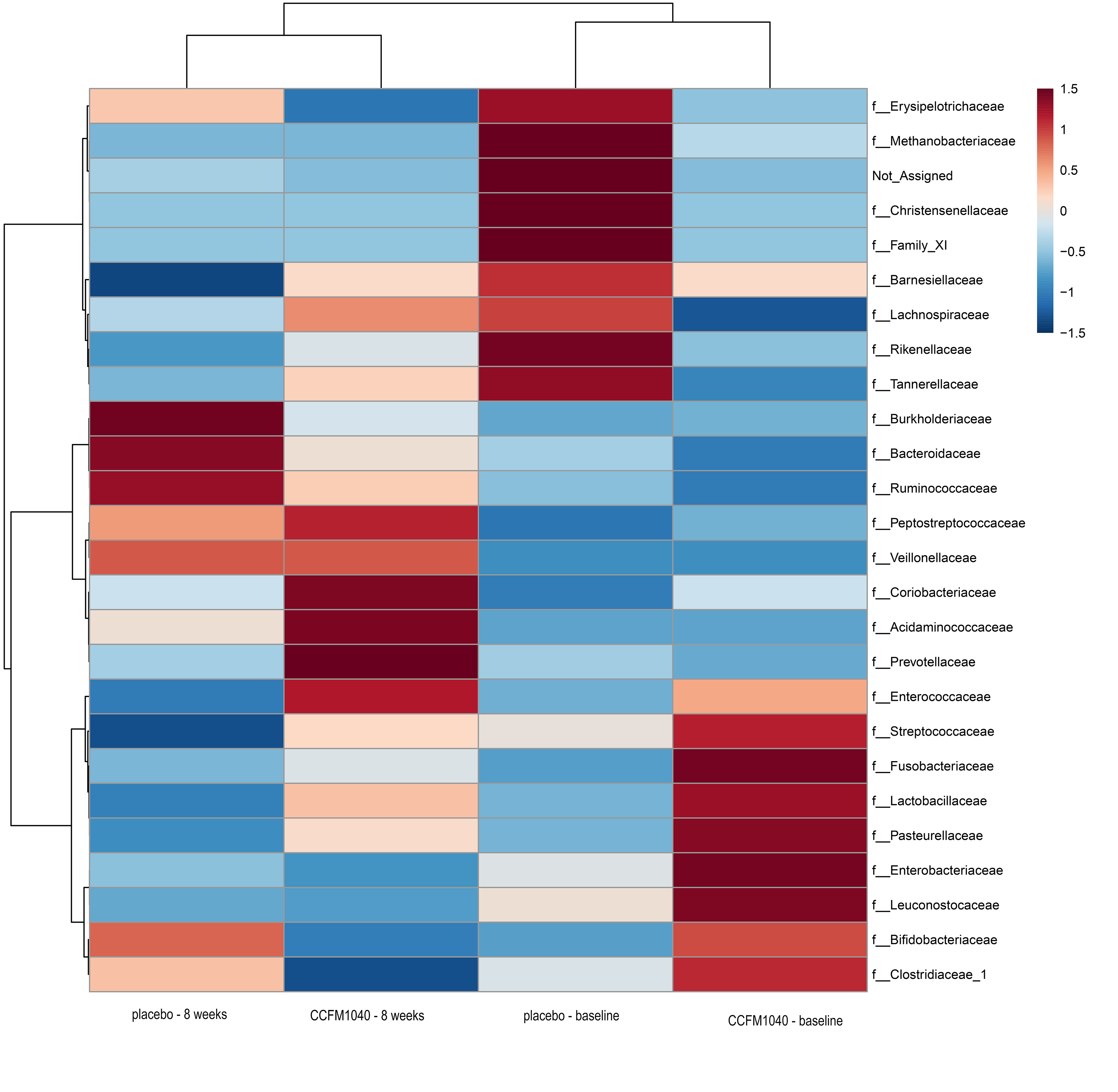

Supplement: Supplementary Figure 3 — Heatmap analysis of family abundance clustering in patients with AR. P < 0.05. The heatmap shows the rank based on abundance. Each column represents one group, while each row in the heatmap represents one family. The red to blue color bar indicates the relative abundance. AR, allergic rhinitis. [file Image_3.TIF]

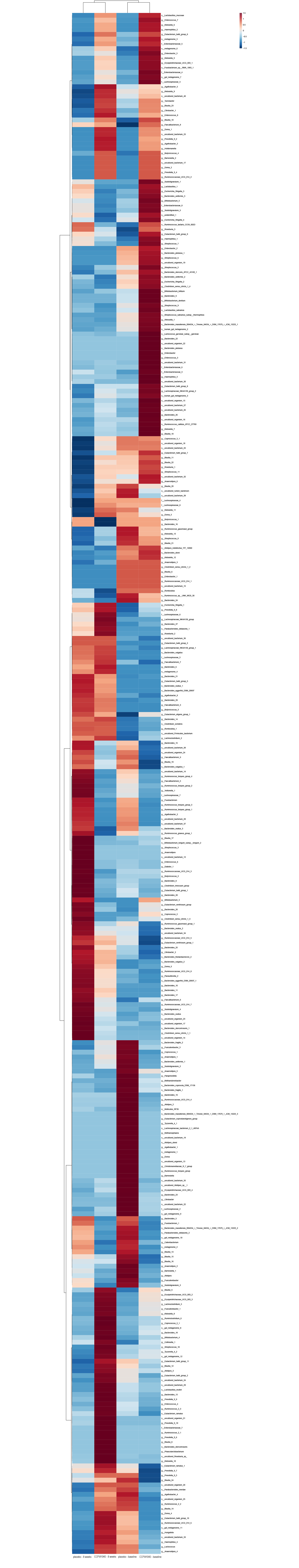

Supplement: Supplementary Figure 4 — Heatmap analysis of feature abundance clustering in patients with AR. P < 0.05. The heatmap shows the rank based on abundance. Each column represents one group, while each row in the heatmap represents one feature. The red to blue color bar indicates the relative abundance. AR, allergic rhinitis. [file Image_4.TIF]
